# Advances in Catatonia: Diagnostic Breakthroughs, Neuroimaging Insights, and Emerging Treatments

**DOI:** 10.1192/j.eurpsy.2025.1857

**Published:** 2025-08-26

**Authors:** M. Peyioti, P. Argitis, A. Karampas, M. Demetriou, V. Anagnostopoulou, Z. Chaviaras

**Affiliations:** 1 University General Hospital of Alexandroupolis, Alexandroupolis; 2 Psychiatric Clinic, General Hospital of Corfu, Corfu; 3 Psychiatric Clinic, University General Hospital of Ioannina, Ioannina, Greece

## Abstract

**Introduction:**

Catatonia is a neuropsychiatric syndrome characterized by motor, behavioral, and autonomic disturbances, frequently associated with psychiatric disorders such as schizophrenia and mood disorders, but also manifesting in medical conditions like autoimmune encephalitis and epilepsy. Despite its prevalence, catatonia remains underdiagnosed and undertreated. Recent advances in neuroimaging, diagnostic frameworks, and therapeutic interventions have enhanced our understanding of the syndrome, contributing to improved clinical outcomes.

**Objectives:**

This abstract synthesizes recent developments in the diagnosis and treatment of catatonia based on research conducted from 2020 to 2024.

**Methods:**

A systematic review of the literature published between 2020 and 2024 was performed using databases such as PubMed and Google Scholar. The search strategy included the terms “catatonia,” “diagnosis,” “treatment,” and “neuroimaging.” Relevant studies, case reports, and reviews focusing on diagnostic innovations, neuroimaging techniques, and therapeutic advances were included.

**Results:**

Advances in neuroimaging, particularly functional MRI, have revealed abnormalities in the frontoparietal cortex, basal ganglia, and cerebellum, offering insights into the neurobiological mechanisms underlying catatonia. Resting-state fMRI studies have demonstrated altered functional connectivity in these regions, facilitating differentiation between catatonia and other movement disorders. Diagnostic tools such as the Bush-Francis Catatonia Rating Scale (BFCRS) remain integral to clinical assessment.

Benzodiazepines, particularly lorazepam, continue to be the first-line treatment, while electroconvulsive therapy (ECT) is employed in treatment-resistant cases, especially in malignant catatonia marked by autonomic dysregulation. Emerging treatments, including NMDA receptor antagonists like memantine, have shown potential in refractory cases. Special considerations are required for populations with autism spectrum disorder (ASD) and neurodevelopmental conditions, where catatonia presents uniquely.

**Conclusions:**

Recent findings underscore the importance of early diagnosis and timely intervention in catatonia. Advances in neuroimaging and novel therapeutic options have enhanced the management of this complex condition, opening new directions for research and clinical practice.

**Disclosure of Interest:**

None Declared

